# Low back pain precedes the development of new knee pain in the elderly population; a novel predictive score from a longitudinal cohort study

**DOI:** 10.1186/s13075-019-1884-0

**Published:** 2019-04-15

**Authors:** Hiromu Ito, Shinjiro Tominari, Yasuharu Tabara, Takeo Nakayama, Moritoshi Furu, Tomotoshi Kawata, Masayuki Azukizawa, Kazuya Setoh, Takahisa Kawaguchi, Fumihiko Matsuda, Shuichi Matsuda, on behalf of the Nagahama Study group, Yasuharu Tabara, Takahisa Kawaguchi, Kazuya Setoh, Yoshimitsu Takahashi, Shinji Kosugi, Takeo Nakayama, Fumihiko Matsuda

**Affiliations:** 1 Department of Orthopaedic Surgery, Kyoto University Graduate School of Medicine, 54 Kawahara-cho, Shogoin, Sakyo, Kyoto, 606-8507 Japan; 2 Department of Health Informatics, Kyoto University Graduate School of Medicine, Konoe-cho, Yoshida, Kyoto, 606-8501 Japan; 3 Center for Genomic Medicine, Kyoto University Graduate School of Medicine, Konoe-cho, Yoshida, Kyoto, 606-8501 Japan

**Keywords:** Knee pain, Predictive score, Risk factor, Mental health, Low back pain

## Abstract

**Background:**

To investigate the association between knee pain and risk factors including low back pain and to develop a score to predict new knee pain in an older population, using population-based longitudinal cohort data.

**Methods:**

We collected a questionnaire on self-reported knee pain and demographic data in a systematic manner from community residents aged ≥ 50 years twice, at baseline, and after 5 years. Multivariate logistic regression analyses were performed to investigate the association between knee pain and risk factors and to build a predictive model that would enable calculation of the risk of the development of knee pain within 5 years. The model is presented in the form of score charts.

**Results:**

A total of 5932 residents aged ≥ 50 years from the cohort of 9764 that completed the first questionnaire were enrolled in the second survey. After exclusions, paired data for the two time points an average of 5.4 years apart were analyzed for 4638 participants. Multivariate analyses showed older age, female sex, higher BMI, weight increase, lower mental health score, and higher back pain/disability score were independent risk factors for knee pain. The predictive score comprised six factors: age, sex, BMI, weight increase, mental health, and low back pain/disability. The risk of developing knee pain ranged from 11.0 to 63.2% depending on the total score.

**Conclusion:**

This study demonstrated a significant association between knee and low back pain/disability along with other risk factors. The score we developed can be used to identify a population without any imaging modality who are at high risk of developing knee pain.

**Electronic supplementary material:**

The online version of this article (10.1186/s13075-019-1884-0) contains supplementary material, which is available to authorized users.

## Background

Knee pain is one of the widespread, disturbing joint symptoms in the older population worldwide, and osteoarthritis (OA) is one of the most common causes of the symptoms. It has been estimated that more than 30% of the general population aged ≥ 50 years suffer from OA of the knee joint [1, 2]. A recent report showed that disability-adjusted life years for knee OA reduced by 2.4% from 2006 to 2016 even after standardization for age, which is much more than the reduction from rheumatoid arthritis or low back and neck pain [3]. Therefore, the pathophysiology and etiology of knee pain have attracted increasing attention and preventive measures have been vigorously pursued, especially in developed countries.

The prevalence of the disease has been estimated using mainly X-rays because of their availability and reliability worldwide. However, this requires an accessible X-ray device, radiological exposure, and a reliable evaluator. It is also well known that a difference exists between the degree of joint destruction judged by X-ray and the actual symptoms in the knee joint; there is a certain percentage of the population whose knees show radiographic OA but who have negligible symptoms, and vice versa. One report even showed that over a period of 20 years the percentage of people who had knee symptoms had increased despite a decrease in the percentage of those with radiographic knee OA [4]. Therefore, it is essential to consider the symptom rather than the radiographic degree of joint destruction when preventive strategies are considered and planned.

The identification of prognostic and risk factors for progression of knee pain and/or clinical knee OA has attracted intensive study, and several such factors have been reported. A meta-analysis showed that age, ethnicity, body mass index (BMI), baseline OA severity, and joint effusion were strongly linked to progression [5]. In addition, several reports have shown that poor mental health is associated with worsening of symptoms [6]. Moreover, the relationship between pain in different parts of the body has also gradually gained attention. Especially, knee pain and low back pain are two of the most frequent, unanimous pain/disabilities in the elder population. It is highly conceivable that one can affect the other. However, the association between knee and low back pain has not been thoroughly investigated. The entire spectrum of risk factors for this association remains ambiguous.

To detect those at risk of knee pain, several scores and formulae have been proposed [7–9]. However, few of these are applicable to people who do not yet have knee pain but who are likely to develop symptoms later. Awareness of factors that are applicable to these individuals is crucial for developing a formula to predict the development of knee pain.

### Study objectives

The aims of this study were to investigate the association between knee and low back pain/disabilities and to develop a predictive score that enables the identification of those who are likely to develop new knee pain within a period of 5 years. We selected participants aged ≥ 50 years because it has been shown that the prevalence of knee OA increases dramatically over the age of 50 [1, 2].

## Materials and methods

### Study participants

This prospective, longitudinal study included part of the general, comprehensive cohort that was recruited from the general population living in Nagahama, a largely rural city of 125,000 inhabitants in Shiga Prefecture, located in central Japan, and which has been reported elsewhere [10, 11]. We recruited residents for this particular study between 2007 and 2010. The inclusion criteria for the study were as follows: (1) aged ≥ 50 years at the time of the first survey, (2) able to participate independently in the health examination, (3) having no difficulties in communication, and (4) voluntarily deciding to participate in the project. This study was designed in accordance with the Helsinki Declaration and was approved by the Ethics Committee of Kyoto University Graduate School and Faculty of Medicine (No. C278). Written informed consent for this study was obtained from all participants.

A total of 5932 people aged ≥ 50 years agreed to participate in this study at the first surveillance from 2007 to 2010. Then, the second survey was sent in 2015 to all respondents of the first survey, and 5576 participants returned the form (94.0% response). The paired data from the two time points were analyzed, and the 5046 subjects whose total pain score of the Japanese Knee Osteoarthritis Measure (JKOM) [12] increased by 3 or more (9% net increase in a possible 32 points) were analyzed as a “new symptom” group, based on a previous similar report [13].

### Assessment of knee pain

The presence of knee pain was determined by a patient-reported outcome score, JKOM, which was established and validated previously [12]. The pain score consists of eight subscales, in each of which, a subject chooses no to severe symptom. No symptom is regarded as score 0, and severe symptom is score 4. Subjects whose scores were 0 or 1 in all the eight pain subscales were included in subsequent analyses as “no symptom” subjects.

### Predictor variables

Basic clinical parameters were measured and surveyed at baseline. Blood and urine samples were also collected. Age at the time of the first survey, sex, and BMI based on height and weight at the first survey were recorded. Information about clinical history, smoking, and drinking habits was obtained using a structured questionnaire. The weight change between the time of the first survey and when the participant was 20 years old was reported by the participant in five categories as no change (< 3 kg), slight increase (3–10 kg), substantial increase (> 10 kg), slight decrease (3–10 kg), and substantial decrease (> 10 kg). Participants were asked whether they were never smokers, had stopped smoking, or were current smokers. Current smokers were asked how many cigarettes they smoked per day and how many years they had smoked. Participants were also asked whether they were never regular drinkers, had stopped drinking, or drank currently. Current drinkers were asked how much they consumed per day using self-calculation of total units, with a unit equaling a bottle of beer (500 ml), a glass of wine (240 ml), or a shot of liquor (180 ml) [14]. Participants were asked to classify how much they moved in daily life as sedentary (mostly sitting), moderately active (sometimes did walking, shopping, or light sport) or active (played sports or did exercise regularly). The severity of low back pain and its disabilities was evaluated by the Roland–Morris disability questionnaire (RMDQ) [15]. Mental health was surveyed using a subscale of the Short-Form 36-Item Health Survey (SF36) [16]. We also measured the ankle-brachial pressure index (ABI) as a marker of vessel aging, serum high-sensitivity C-reactive protein (hsCRP) as a marker of inflammation, and urine cross-linked *N*-telopeptide of type I collagen (NTX) and urine C-terminal telopeptide of type I collagen (CTX) as markers of osteoporosis.

### Statistical analyses

To conduct multivariate analyses, we divided the entire group into the two for mental health and low back pain by the median score, respectively. We performed logistic regression analysis to calculate the regression coefficients of the predictor variables. The backwards stepwise selection method was used to reduce the number of predictors incorporated into the final model, the aim being model simplicity. The significance level for removing variables from the model was set at *P* ≥ 0.20. We employed a scoring system to present the final model. Each predictor regression coefficient was divided by twice the smallest regression coefficient and rounded to the nearest integer. We calculated the risk of pain worsening at 5 years as *e*^lp^/(1 + *e*^lp^)*,* where lp is the linear predictor for each subject. Prediction model made by regression analysis is known to overfit the data and have problem in terms of generalizability. Therefore, we applied a shrinkage factor to the regression coefficients when calculating risk of knee pain worsening for better prediction (Additional file 1: Supplementary note) [17, 18]. To measure the performance of the model, we used the C-index to assess its discriminatory ability. We also evaluated the calibration by plotting the predicted risk in deciles against the corresponding proportion of the subjects who experienced worsening of knee pain.

All other tests were two-sided, with *P* < 0.05 considered significant. Statistical analyses were performed using Stata/IC software, version 14.2 (StataCorp LLC, College Station, TX, USA).

## Results

### Description of cohort

**Fig. 1:**
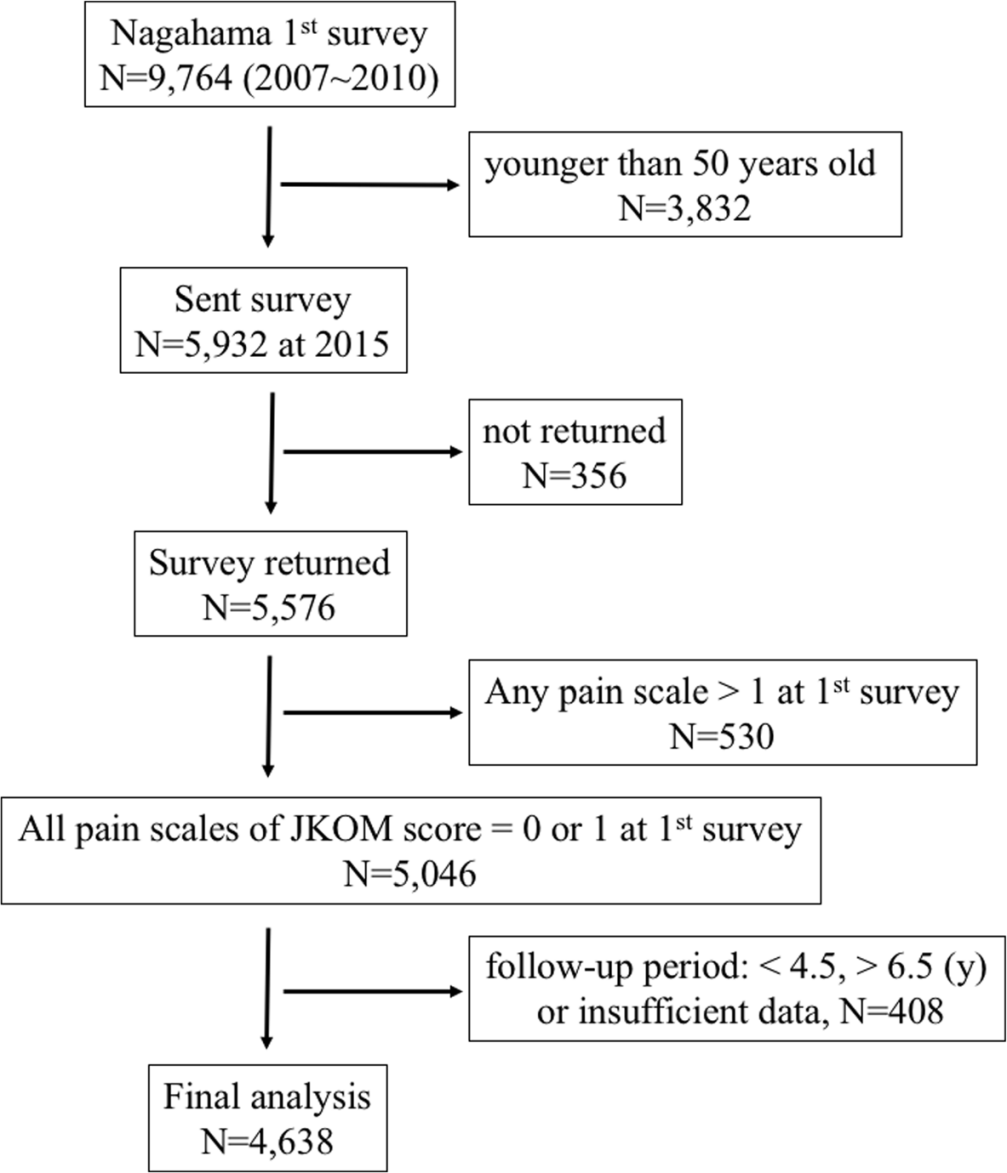
Participant flow diagram showing the selection of subjects for the study

Figure 1 shows the process for selection of the participants. As planned, we selected the 5046 respondents with negligible knee pain (90.5%). The data for participants whose follow-up period was < 4.5 or > 6.5 years were excluded from further analyses (408, 8.1%), resulting in 4638 participants being analyzed. The average follow-up period was 5.4 years (4.8–6.2 years).

At baseline, the average age of the participants was 62.4 years, and the majority were women (64.8%). Their average BMI was 22.5, which was comparable to that reported elsewhere in Japan [19]. The averages of the JKOM were 1.1 ± 2.1 [0–8] at baseline and 2.8 ± 4.1 [0–30] at the follow-up. The detailed demographic data are shown in Table 1. The demographic data of non-responders (*n* = 356) were not significantly different from those of responders (data not shown).

**Table 1:** Demographic data (*n* = 4638)

Variables	With new knee pain (*n* = 1262)	Without new knee pain (*n* = 3376)
Age (year)	63.6 ± 6.5 [50–75]	62.0 ± 6.3 [50–75]
Sex		
Male	411 (32.6)	1221 (36.2)
Female	851 (67.4)	2155 (63.8)
BMI	23.1 ± 3.1 [14.3–36.1]	22.3 ± 2.8 [13.6–37.8]
Weight change, No (%)		
Within 3 kg	286 (22.7)	965 (28.6)
3〜10 kg increase	473 (37.5)	1190 (35.3)
10 kg < increase	277 (22.0)	592 (17.5)
3〜10 kg decrease	226 (17.9)	629 (18.6)
3 kg < decrease	0 (0)	0 (0)
Smoking		
Current smoker	130 (10.3)	361(10.7)
Quitted	263 (20.8)	728 (21.6)
Never smoker	869 (68.9)	2287 (67.7)
Alcohol		
Current drinker	688 (54.5)	2034 (60.1)
Quitted	31 (2.5)	46 (1.4)
Never drinker	543 (43.0)	1296 (38.4)
Alcohol consumption (unit/day)		
< 1 unit	420 (61.0)	1200 (59.3)
1 ≤ consumption < 2	198 (28.7)	591 (29.2)
2 ≤ consumption < 3	62 (9.0)	182 (9.0)
3 ≤ consumption	9 (1.3)	49 (2.5)
Activity		
Sedentary	76 (6.0)	237 (7.0)
Moderately active	883 (70.0)	2341 (69.3)
Active	303 (24.0)	798 (23.7)
Mental health (points)	18.5 ± 3.4 [6–25]	19.1 ± 3.3 [5–25]
Low back pain (points)	0, 0–1 [0–20]	0, 0–2 [0–20]
ABI	1.09 ± 0.08 [0.58–1.31]	1.08 ± 0.07 [0.51–1.39]
hsCRP (mg/dl)	1019.2 ± 3073.5 [50–52,700]	897.1 ± 3338.4 [50–1,260,000]
NTX (nmolBCE/nmolCr)	40.5 ± 21.0 [7–154]	40.2 ± 20.2 [5–196]
CTX (μg/nmolCr)	224.9 ± 139.1 [14–1005]	226.4 ± 131.0 [13–1103]

Data for continuous variables are expressed as mean ± SD [minimum–maximum] except for low back pain (median, inter-quartile range [minimum–maximum]), and data for categorical variables are expressed as numbers and percentages

*BMI* body mass index, *ABI* ankle-brachial pressure index, *hsCRP* high-sensitivity C-reactive protein, *NTX* cross-linked *N*-telopeptide of type I collagen (urine), *CTX* cross-linked C-telopeptide of type I collagen (urine)

### Logistic regression modeling

A total of 1262 participants whose total score worsened by 3 or more points in the second survey were identified. Univariate analysis was performed to identify relevant factors and showed that greater age, female sex, higher BMI, weight increase, heavy drinking, worse mental health, and the presence of low back pain/disability were significant factors in the development of knee pain (data not shown). Multivariate analysis identified the same risk factors that were identified in univariate analysis, except for heavy drinking (Table 2, model 1). Then, multivariate analysis with stepwise selection identified six factors that were risk factors for the incidence of knee pain (Table 2, model 2). Furthermore, low back pain/disability was statistically significant even if it was treated as a continuous variable; odds ratios were 1.10 (95% CI 1.08–1.13) in univariate analysis and 1.08 (95% CI 1.06–1.11) in multivariate analysis.

**Table 2:** Multivariate analysis

	Model 1 (*n* = 4482)			Model 2 (*n* = 4638)			
	Odds ratio	95% CI	*P* values	Odds ratio	95% CI	*P* values	Regression coefficient
Age							
Fifties	Reference	–	–	Reference	–	–	
Sixties	1.54	1.31–1.81	< 0.001	1.54	1.32–1.80	< 0.001	0.431
Seventies	2.40	1.94–2.96	< 0.001	2.35	1.91–2.88	< 0.001	0.852
Sex							
Male	Reference	–	–	Reference	–	–	
Female	1.42	1.13–1.80	0.003	1.34	1.16–1.55	< 0.001	0.294
BMI							
< 25	Reference	–	–	Reference	–	–	
25≤	1.67	1.41–1.99	< 0.001	1.66	1.40–1.96	< 0.001	0.504
Weight change							
No change	Reference	–	–				
3 kg ≤ increase	1.26	1.06–1.50	0.008	1.27	1.08–1.50	0.005	0.240
3 kg ≤ decrease	1.09	0.88–1.34	0.42	1.10	0.90–1.35	0.36	0.096
Alcohol consumption (unit per day)							
No or occasional drinker	Reference	–	–				
1 ≤ consump. < 2	0.89	0.72–1.09	0.26				
2 ≤ consump. < 3	0.96	0.69–1.34	0.82				
3≤	0.48	0.23–1.01	0.054				
Smoking							
Never smoker	Reference	–	–				
Quitted	1.30	0.99–1.71	0.055				
Current smoker	1.14	0.91–1.44	0.26				
Activity							
Moderately active	Reference	–	–				
Sedentary	0.90	0.68–1.19	0.46				
Active	1.10	0.94–1.29	0.24				
Mental health (points)							
20≤	Reference	–	–	Reference	–	–	
≤ 19	1.34	1.21–1.59	< 0.001	1.34	1.21–1.59	< 0.001	0.325
Low back pain (points)							
0	Reference	–	–	Reference	–	–	
1≤	1.62	1.40–1.86	< 0.001	1.59	1.38–1.83	< 0.001	0.463
ABI							
1≤	Reference	–	–				
0.9 ≤ ABI < 1	0.90	0.71–1.12	0.34				
< 0.9	0.73	0.42–1.25	0.25				
hsCRP	1.00	1.00–1.00	0.69				
NTX	1.00	0.99–1.01	0.80				
CTX	1.00	1.00–1.00	0.80				

*95% CI* 95% confidence interval, *BMI* body mass index, *ABI* ankle-brachial pressure index, *hsCRP* high-sensitivity C-reactive protein, *NTX* cross-linked *N*-telopeptide of type I collagen (urine), *CTX* cross-linked C-telopeptide of type I collagen (urine)

### Development of a predictive score

Based on these analyses, we developed a predictive score (Table 3). The total possible score is 14, as outlined above, consisting of 4 points for age, 2 points for female sex, 3 points for BMI, 1 point for weight increase, 2 points for mental health, and 2 points for low back pain. The risk of developing new knee pain ranged from 11.0 to 63.2% depending on the total score (Table 4). The model calibration was good, with close agreement between the predicted and observed incidence of new knee pain (Additional file 2: Figure S1). The calculated C-index was 0.6326. The scores 2 and 4 of age, 2 of sex, 3 of BMI, 2 of mental health, 2 of low back pain, and 1 of weight increase were attributed to this population of 51.5%, 15.8%, 64.8%, 19.3%, 52.5%, 30.3%, and 54.6%, respectively. The score was normally distributed in this population (Additional file 3: Table S1).

**Table 3:** Prediction score

	Score
Age	
< 60	0
60 ≤ age < 70	2
70≤	4
Sex	
Male	0
Female	2
BMI	
< 25	0
25≤	3
Mental health (points)	
20≤	0
≤ 19	2
Low back pain (points)	
0	0
1≤	2
Weight change (compare with 20 years old)	
Within 3 kg	0
3 kg ≤ increase	1
3 kg ≤ decrease	0

*BMI* body mass index

**Table 4:** Prediction probability

Total score	Probability	95% CI
0	11.0%	8.9–13.6%
1	13.0%	10.8–15.5%
2	14.7%	12.3–17.5%
3	17.5%	15.0–20.2%
4	19.6%	16.7–23.0%
5	23.1%	20.1–26.4%
6	26.3%	22.6–30.4%
7	30.3%	26.6–34.3%
8	34.0%	29.5–38.7%
9	39.0%	34.3–43.8%
10	42.2%	37.2–47.3%
11	48.3%	42.6–54.2%
12	51.3%	46.0–56.6%
13	56.5%	49.6–63.2%
14	60.8%	54.9–66.3%

*95% CI* 95% confidence interval

## Discussion

This longitudinal study of 4638 participants (78.2% of the possible participants) in the general population showed that older age, female sex, higher BMI, weight increase, lower mental health score, and higher low back pain/disability score were significant risk factors for developing new knee pain in people aged ≥ 50 years. We developed a predictive score that showed that the risk of developing new knee pain within 5 years ranged from 11.0 to 63.2% depending on the total score. This is the first study to show the effect of low back pain/disability and other risk factors on the risk of developing new knee pain and to develop a reliable, easy-to-use predictive score.

In general, the association between knee and low back pain/disabilities has not been well studied. Muraki et al. reported that knee pain and low back pain were significantly associated with the magnitude of quality of life loss in 1369 women aged ≥ 40 years in the general population [20]. However, they did not show a direct association between the two symptoms nor analyze the predictive value of low back pain. We previously reported that combined knee and low back pain additively strengthened the correlation with sleep problems, but a direct association between the two types of pain was not shown [10]. The current study clearly illustrates this association, because in univariate analysis, the presence of low back pain/disability scored at just 1 point increased the risk of new knee pain 1.6 times, which was a greater effect than female sex or weight increase and a similar effect of higher BMI, three of the known risk factors. Furthermore, a previous report of musculoskeletal pain showed that knee pain had poorer outcomes compared with low back pain, indicating that it was a constant burden in the daily life of older people [21].

One of possible pathophysiological mechanisms of this association is that osteoarthritic pathology can affect any joints or body parts in the older population, especially, load-bearing organs such as the knee and the lumbar vertebrae. From a clinical point of view, it is not exactly known how preceding low back pain/disability can predict new knee pain, but it is conceivable that one tends to affect the other by worsening the load-bearing burden of the other and/or by loosening the balance of the body when walking and even standing. Indeed, it was shown that the number of painful sites outside the knee, including low back pain, independently predicted knee cartilage volume loss without knee OA [22, 23], which indicates the crucial association between musculoskeletal pain at different sites. Pain is one of the central issues in the management of knee OA [24–26], and this association should be investigated in future studies.

Numerous reports have identified several risk factors for knee pain or knee OA. A meta-analysis showed strong evidence for age, ethnicity, BMI, co-morbidity count, joint effusion, and baseline severity as risk factors [5]. The results of the present study support those of the meta-analysis, identifying age and BMI as strong risk factors. In contrast, smoking and alcohol consumption were not significant risk factors in our study, which is also consistent with previous reports [12, 27]. We did not find any significant differences in risk between people with different levels of daily activity, although the current consensus would be that exercise and an active daily life contribute to reducing the possibility of knee OA [26]. A possible explanation for this difference is that the current study used a simple question to evaluate activity with the responses sedentary, moderately active, or active, and could not define how each participant lived their daily life and how much they moved or exercised. More detailed collection of data may detect differences.

Several meta-analyses and reviews have shown that metabolic syndrome and dyslipidemia are risk factors for knee OA [28–30], and a large-scale study has also shown that OA is a significant risk factor for cardiovascular diseases [31]. Therefore, we decided to include the ABI, which is a reliable, objective measurement of peripheral artery disease [32]. Contrary to our expectations, no significant association between the development of knee symptoms and ABI was apparent, possibly because ABI alone is not sufficient to predict new knee symptoms, or because knee symptoms, unlike radiographic OA, may not be directly related to vascular manifestations such as arteriosclerosis. In addition, a previous report showed that hsCRP was strongly associated with all definitions of radiographic OA [33]. However, that study also showed that the association was not independent of BMI, and our data support the notion that hsCRP is not an independent risk factor for new knee symptoms. Similarly, a previous study showed that the radiographic features of OA are associated with bone mineral density of the lumbar spine and femoral neck [34]. However, NTX and CTX, two reliable biomarkers of osteoporosis, failed to predict new knee symptoms. It is reasonable to assume that these current osteoporosis biomarkers alone, or possibly osteoporosis itself, are insufficient to predict the development of knee symptoms. It may be necessary to collect more detailed information about osteoporosis to identify any contribution to the development of knee symptoms.

The production of models to predict the development of radiographic knee OA or knee symptoms has been vigorously pursued. However, most previous studies report only the odds ratios of certain risk factors or the results of statistical models such as Cox proportional hazards models, and the process for selecting people at risk remains ambiguous. Kerkhof et al. reported a predictive model for knee OA incidence including clinical, genetic, and biochemical risk factors [8]. However, gene analysis requires reliable access to a competent analytical department and is not suitable for screening of people at risk. Zhang et al. reported a simple predictive score using age, BMI, and scores defined relative to an index person [7], but that score also requires analysis of knee radiography, which necessitates radiological exposure and a reliable evaluator. Fernandes et al. recently reported a useful, simple predictive model using only self-reported predictors without any imaging studies or laboratory data [9]; however, their calculation is rather complex and requires certain stratagems to obtain an individual risk. The current study shows that a self-reported score without any invasive tests can be sufficient to select people at risk with a desirable probability. The actual potential of the developed score should be verified in the future.

### Limitations of the study

Nevertheless, this study involves some unavoidable limitations. First, the origin of knee symptoms was not confirmed by any method. Knee symptoms may be confused with lower leg pain originating from back ailments, although the JKOM questionnaire is designed to elicit knee-specific symptoms. Second, we did not collect any imaging data, which would increase the reliability of the score. However, the purpose of the current study was to develop a reliable score that did not require any invasive measurements, and any imaging studies should be used in a different setting. Third, our analyses were performed with a predetermined set of data. We cannot exclude the existence of other factors that could contribute to the prediction of new knee symptoms, including injury history, educational level, and metabolic syndrome. Fourth, the threshold of the score determining the need for appropriate intervention is unclear, and this should be extensively studied in a future longitudinal, proof-of-concept study. Finally and importantly, prediction score constructed by regression analysis tends to overfit the data which the score was derived from, especially when using stepwise selection. Therefore, the performance of our score needs to be confirmed by external patient data.

## Conclusions

A total of 4638 participants completed the two surveys of knee symptoms at an average interval of 5.4 years and were analyzed. Multivariate analyses showed that older age, female sex, higher BMI, lower mental health score, weight increase, and higher low back pain/disability score were significant risk factors for the development of new knee pain in people aged ≥ 50 years who had no or negligible knee symptoms. We developed a predictive score including low back pain/disability score that indicated that the risk of developing new knee pain within 5 years ranged from 11.0 to 63.2%, depending on the total score.

## Additional files

Additional file 1: Supplementary note. (DOCX 13 kb)

Additional file 2: **Figure S1.** The association between the average of predicted probability and observed probability. Dots indicate the relationship between mean predicted risk of developing knee pain in deciles and corresponding observed risks. Diagonal dashed line indicates perfect concordance between predicted and observed risk of developing knee pain. (TIFF 2197 kb)

Additional file 3: **Table S1.** Distribution of score of this population (DOCX 18 kb)

## Abbreviations

ABI: Ankle-brachial pressure index
BMI: Body mass index
CTX: Urine C-terminal telopeptide of type I collagen
hsCRP: Serum high-sensitivity C-reactive protein
JKOM: Japanese Knee Osteoarthritis Measure
NTX: Urine cross-linked *N*-telopeptide of type I collagen
OA: Osteoarthritis
RMDQ: The Roland–Morris disability questionnaire
SF36: The Short-Form 36-Item Health Survey
