# Supplementary material for: Low back pain precedes the development of new knee pain in the elderly population; a novel predictive score from a longitudinal cohort study
Source: Arthritis Res Ther. 2019 Apr 15;21:98. doi: 10.1186/s13075-019-1884-0 (PMC6466785; doi:10.1186/s13075-019-1884-0)
Supplement: Supplementary file 1 — Supplementary note. (DOCX 13 kb) [file 13075_2019_1884_MOESM1_ESM.docx]

Supplementary note.

We calculated the risk of pain worsening at five years as *e^lp^*/(1+*e^lp^*)*,* where *lp* is the linear predictor for each subject. When calculating linear predictor *lp*, uniform shrinkage factor *s*

*s* = (model χ^2^ – *df*) / model χ^2^ = (202.26-8)/202.26　= 0.960

is applied (*df*: degree of freedom). Thus;

*lp* = 0.960 × { 0.431 (if 60≦age<70)+ 0.852 (if 70≦age<80)+ 0.294 (if female)+ 0.504 (if BMI≧25)+ 0.325 (if mental health≦19)+ 0.463 (if low back pain≧1)+ 0.240 (if weight gain≧3kg)+ 0.096 (if weight loss≧3kg) }
