# Supplementary figures and images for: Low back pain precedes the development of new knee pain in the elderly population; a novel predictive score from a longitudinal cohort study
Source: Arthritis Res Ther. 2019 Apr 15;21:98. doi: 10.1186/s13075-019-1884-0 (PMC6466785; doi:10.1186/s13075-019-1884-0)

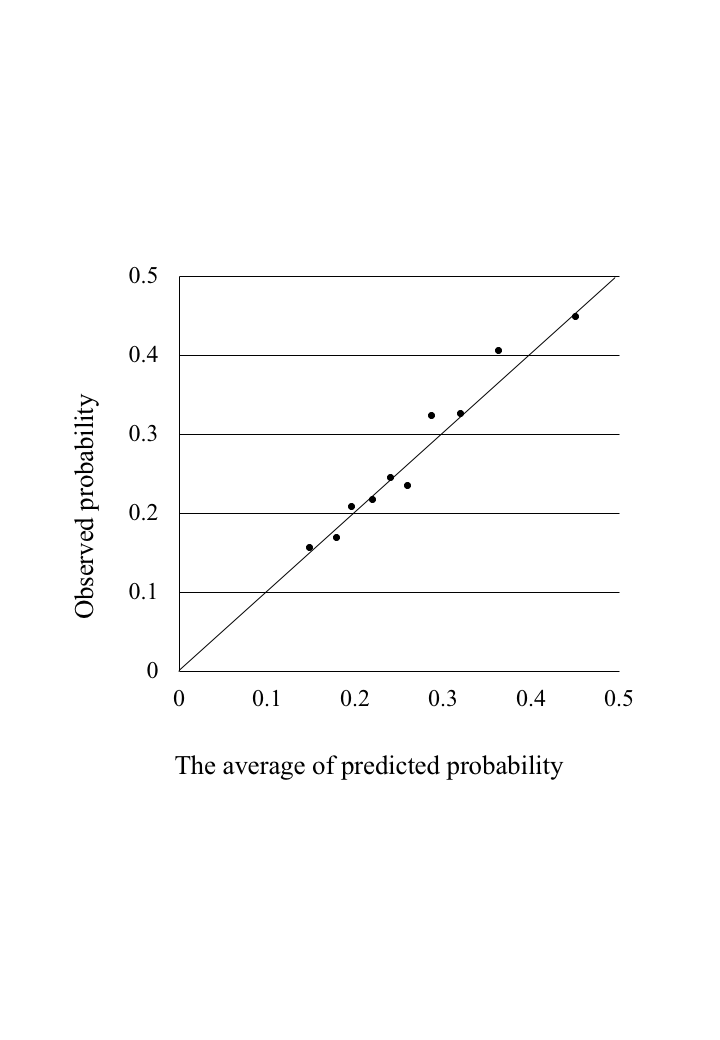

Supplement: Supplementary file 2 — Figure S1. The association between the average of predicted probability and observed probability. Dots indicate the relationship between mean predicted risk of developing knee pain in deciles and corresponding observed risks. Diagonal dashed line indicates perfect concordance between predicted and observed risk of developing knee pain. (TIFF 2197 kb) [file 13075_2019_1884_MOESM2_ESM.tiff]
