# Supplementary material for: Low back pain precedes the development of new knee pain in the elderly population; a novel predictive score from a longitudinal cohort study
Source: Arthritis Res Ther. 2019 Apr 15;21:98. doi: 10.1186/s13075-019-1884-0 (PMC6466785; doi:10.1186/s13075-019-1884-0)
Supplement: Supplementary file 3 — Table S1. Distribution of score of this population (DOCX 18 kb) [file 13075_2019_1884_MOESM3_ESM.docx]

Table S1 Distribution of score of this population

| Total score | n | percentage |
| --- | --- | --- |
| 0 | 21 | 0.5 |
| 1 | 48 | 1.0 |
| 2 | 354 | 7.6 |
| 3 | 374 | 8.1 |
| 4 | 779 | 16.8 |
| 5 | 683 | 14.7 |
| 6 | 774 | 16.7 |
| 7 | 509 | 11.0 |
| 8 | 513 | 11.1 |
| 9 | 203 | 4.4 |
| 10 | 247 | 5.3 |
| 11 | 38 | 0.8 |
| 12 | 75 | 1.6 |
| 13 | 5 | 0.1 |
| 14 | 15 | 0.3 |
